# Production of high concentrated cellulosic ethanol by acetone/water oxidized pretreated beech wood

**DOI:** 10.1186/s13068-017-0737-9

**Published:** 2017-02-28

**Authors:** Constantinos Katsimpouras, Konstantinos G. Kalogiannis, Aggeliki Kalogianni, Angelos A. Lappas, Evangelos Topakas

**Affiliations:** 1 Industrial Biotechnology & Biocatalysis Group, School of Chemical Engineering, National Technical University of Athens, 9 Iroon Polytechniou Str., Zografou Campus, 15780 Athens, Greece; 2 Chemical Process and Energy Resources Institute (CPERI), CERTH, 6th km Harilaou-Thermi Road, Thermi, 57001 Thessalonica, Greece

**Keywords:** Beech wood, Wet oxidation, Ethanol fermentation, Enzymatic liquefaction, High gravity

## Abstract

**Background:**

Lignocellulosic biomass is an abundant and inexpensive resource for biofuel production. Alongside its biotechnological conversion, pretreatment is essential to enable efficient enzymatic hydrolysis by making cellulose susceptible to cellulases. Wet oxidation of biomass, such as acetone/water oxidation, that employs hot acetone, water, and oxygen, has been found to be an attractive pretreatment method for removing lignin while producing less degradation products. The remaining enriched cellulose fraction has the potential to be utilized under high gravity enzymatic saccharification and fermentation processes for the cost-competing production of bioethanol.

**Results:**

Beech wood residual biomass was pretreated following an acetone/water oxidation process aiming at the production of high concentration of cellulosic ethanol. The effect of pressure, reaction time, temperature, and acetone-to-water ratio on the final composition of the pretreated samples was studied for the efficient utilization of the lignocellulosic feedstock. The optimal conditions were acetone/water ratio 1:1, 40 atm initial pressure of 40 vol% O_2_ gas, and 64 atm at reaction temperature of 175 °C for 2 h incubation. The pretreated beech wood underwent an optimization step studying the effect of enzyme loading and solids content on the enzymatic liquefaction/saccharification prior to fermentation. In a custom designed free-fall mixer at 50 °C for either 6 or 12 h of prehydrolysis using an enzyme loading of 9 mg/g dry matter at 20 wt% initial solids content, high ethanol concentration of 75.9 g/L was obtained.

**Conclusion:**

The optimization of the pretreatment process allowed the efficient utilization of beech wood residual biomass for the production of high concentrations of cellulosic ethanol, while obtaining lignin that can be upgraded towards high-added-value chemicals. The threshold of 4 wt% ethanol concentration that is required for the sustainable bioethanol production was surpassed almost twofold, underpinning the efficient conversion of biomass to ethanol and bio-based chemicals on behalf of the biorefinery concept.

**Electronic supplementary material:**

The online version of this article (doi:10.1186/s13068-017-0737-9) contains supplementary material, which is available to authorized users.

## Background

Lignocellulosic biomass feedstocks have garnered a lot of interest, as they constitute a profuse resource for production of biofuels and other high-added-value bio-based materials. Biofuel production from lignocellulosic biomass, such as agricultural or forestry residues, via enzymatic pathways mainly comprises pretreatment, enzymatic saccharification, and fermentation. Pretreatment stands to be the first step to overcome the complexity and recalcitrance of lignocellulosic biomass, rendering cellulose vulnerable to enzymatic hydrolysis [1].

The pretreatment of lignocellulosic biomass is also the costliest part of the process for the production of biofuels. Lignin surrounds cellulose and hemicellulose, essentially making biomass highly recalcitrant to pathogens, microorganisms, and enzymes [2]. One of the pretreatments that have been investigated in the past is the hot compressed water (HCW) treatment also known as hydrothermal treatment, thermohydrolysis, and autohydrolysis. The main aim is to hydrolyze and remove hemicellulose, so as to enhance the fermentability of the biomass and efficiency of the enzymatic processes. Zhu et al. reported that the hemicellulose hydrolysis resulted in pore size and substrate-specific surface increase, thus facilitating the access of cellulase on the cellulose structure [3]. It has also been shown that removing the acetyl groups found on hemicellulose chains can enhance the enzymatic hydrolysis yields of the substrate [4]. However, during hydrolysis of hemicellulose into monosaccharides, there is the simultaneous cleavage of beta-O-4 linkages and b-ethers bonds of lignin and lignin-hemicellulose bonds resulting in the release of phenolic compounds and lignin oligomers that are inhibitors for the downstream enzymatic processes [5, 6]. Therefore, removing them along with the lignin that enhances the recalcitrance of biomass towards enzymes can greatly benefit the fermentation of the resulting substrates.

Among the pretreatment methods that have attracted interest lately are the organosolv processes, which employ organic solvents for removal of the lignin fraction. A wide variety of processes, solvents, and parameters have been investigated ranging from the standard Milox process to combining chemical and physicomechanical pretreatment methods [7, 8]. The Milox process involves delignifying the biomass by treating it with formic and/or acetic acid coupled with hydrogen peroxide so as to produce highly oxidative peroxy acids that cleave the lignin bonds and depolymerize it. The main advantages of these methods are that the solvents and materials can be recovered and reused, and degradation of the dissolved fractions is minimized allowing for their use for production of high-added-value chemicals, such as phenols and hydroxymethylfurfural. In addition, the produced pulps are more easily fermented reducing the overall biofuel production process cost.

Wet oxidation of biomass employing hot water, alkali, and oxygen has also been found to be an interesting pretreatment method. Compared to steam explosion, it has been found to produce much less degradation products, such as 2-furfural and 5-hydroxy methyl-2-furfural compounds, that are well-known inhibitors of microbial growth [9, 10]. As a further development, lately a new process of acetone/water oxidation (AWO) has been developed. In this process, an acetone/water mixture is used instead of water, without alkali use. Very few papers report the effect of this treatment on biomass, but it appears to combine the advantages of wet oxidation such as low temperature and low yield of degradation products in one stage process while achieving much higher delignification of the biomass. Gong et al. reported that the AWO proved to be the most selective in delignifying both sugar maple and hot water extracted sugar maple [10]. The same group successfully delignified *Paulownia* spp. wood with the same method, achieving degrees of delignification (DD) up to 93.6% in a single-stage AWO. They also found that the lignin produced was of high quality, containing no sulfur or inorganic compounds typically found in Kraft produced lignin. Jafari et al. used a mixture of 50 vol% acetone–water solution containing 0.1 wt% of H_2_SO_4_ rather than O_2_, and the yield of enzymatic hydrolysis was improved to 94.2% [11]. The use of acetone and water, two easily separable and recyclable solvents, allows for the development of a low energy intensive, low-cost, green process. Furthermore, to reduce energy demands, such as the distillation energy cost, a fermentation broth exhibiting high ethanol concentration is considered to be a prerequisite and the utilization of high-solids loading of pretreated biomass in the process seems to be the key [12].

High gravity (HG) saccharification and fermentation stand to be a challenging but yet crucial strategy for a cost-competing bioethanol production process. An economically feasible lignocellulosic biomass to bioethanol process is reported to require, among others, a concentration of at least 4 wt% ethanol [13, 14]. However, operating under high initial dry matter (DM) faces many challenges, mainly, due to mass transfer limitations and enzyme inhibition. The conventional stirring techniques result in inadequate mixing, preventing lignocellulolytic enzymes from interacting efficiently with the substrate, while increased end-product inhibition by sugars released during enzymatic hydrolysis leads to low saccharification yields [15]. Alternative mixing systems, such as free-fall mixing, in combination with simultaneous saccharification and fermentation (SSF) have been proved to alleviate the issues related to HG conditions in several cases [16–19].

In the present investigation, the acetone/water oxidized pretreatment of beechwood has been employed for the efficient production of cellulosic ethanol. The pretreatment conditions were optimized by studying the effect of pressure, reaction time, temperature, and acetone-to-water ratio on the final composition of the pretreated samples, as well as in their potential for the enzymatic release of fermentable sugars. The optimized pretreatment conditions were applied for the utilization of beech wood towards the enzymatic liquefaction and saccharification at high initial solids content (20 wt%).

## Results and discussion

### Effect of different AWO conditions on the final composition of the pretreated samples

#### Biomass pretreatment with acetone/water mixtures

The experimental conditions of each run are presented in Table 1, while Table 2 presents the lignin, cellulose, and hemicellulose contents of the final pulp along with recoveries in the solid product for each constituent. It should be noted that in some cases, the recoveries of the constituents are calculated at above 100%, due to the experimental errors of the analytical methods. To understand the effect that acetone and water have on biomass, these two solvents were tested separately both under an inert and an O_2_ rich atmosphere presented in runs 1 through 5 of Table 1. Run no. 1 is essentially a hydrothermal treatment of the biomass at 175 °C. As expected, the main effect of the treatment was on the hemicellulose content, which decreased to around 3.9%. Hemicellulose was extracted and hydrolyzed to oligo- and monomers, with a consequent release of acetic acid, which originates from the cleavage of acetyl groups of the oligosaccharides. Cellulose is mostly unaffected due to its higher crystallinity [20]. The reduction of hemicellulose resulted in an increase in the cellulose and even more in the lignin content of the pretreated biomass. Run no. 2 applied the same experimental conditions as run no. 1 with the exception that a 40 vol% O_2_ rich gas was used instead of N_2_. Hemicellulose was again significantly hydrolyzed, with only around 10 wt% of the original hemicellulose remaining in solid form. The use of an oxidative atmosphere did not affect the lignin content of the pulp, possibly due to the longer reaction time (2 h, compared to 5–30 min typically used in wet oxidation treatment [8]), which allowed the lignin repolymerization and condensation on wood particles.

**Table 1:** Αcetone/water oxidation experimental conditions

Run no.	Acetone/water (wt/wt) ratio	Partial O_2_ pressure at reaction *T* (atm)	Reaction *T* (°C)	Reaction time (h)^a^	Pressure at 20 °C (atm)	Pressure at reaction *T* (atm)
1	0	0	175	2	8.5	20.1
2	0	7.6	175	2	8.5	19.1
3	1:0	0	175	2	8.5	22.4
4	1:0	4.4	175	2	8.5	22
5	1:0	8.7	175	2	8.5	21.8
6	1:1	0	175	2	8.5	27.3
7	1:1	9.9	175	2	8.5	24.9
8	3:1	10	175	2	8.5	25
9	3:1	23.2	175	2	40	58
10	1:1	25.6	175	2	40	64
11	1:3	25.6	175	2	40	64
12	3:1	29.6	200	1	40	74
13	3:1	31.2	200	0.5	40	78
14	3:1	55.6	225	0.5	40	139
15	1:1	9.7	175	2	8.5	24.3
16	3:1	10.1	175	2	8.5	25.4

^a^Heating up and cooling times were each approximately 15 min, which are not included in the reaction time

**Table 2:** Pulp composition and total solids, lignin, hemicellulose, and cellulose recoveries after AWO

Run no.	Exp. Cond.^a^	Constituents in pulp (wt%)					Constituents recovery in solid pulp (wt%)			
		AIL	ASL	Total lignin	Cellulose	Hemicellulose	Total pulp	Lignin	Hemicel.	Cellulose
Beechwood^b^		21.5	2.7	24.2	43.1	20.2				
1	0, 0, 175, 2, 20	33.7	1.7	35.4	59.7	3.9	67.5	98.8	12.9	93.5
2	0, 8, 175, 2, 19	36.4	1.4	37.8	58.7	3.2	65.8	103.1	10.3	89.7
3	1:0, 0, 175, 2, 22	22.8	3.5	26.3	46.6	21.8	94.7	103.0	102.4	102.0
4	1:0, 4, 175, 2, 22	21.8	3.4	25.2	47.5	22.1	92.9	96.9	102.2	101.6
5	1:0, 9, 175, 2, 22	21.4	3.2	24.6	47.1	22.2	93.4	95.2	102.4	102.2
6	1:1, 0, 175, 2, 27	17.6	2.1	19.7	64.6	15.7	69.9	56.8	54.3	104.8
7	1:1, 10, 175, 2, 25	11.1	1.7	12.8	74.8	16.7	59.3	31.4	49.1	103.0
8	3:1, 10, 175, 2, 25	13.9	2.3	16.2	57.0	23.8	73.3	49.0	86.3	97.1
9	3:1, 23, 175, 2, 58	3.2	1.5	4.7	75.2	16.9	50.3	9.7	41.9	87.8
10	1:1, 25, 175, 2, 64	1.0	1.2	2.2	85.9	10.8	45.9	4.2	24.5	91.6
11	1:3, 25, 175, 2, 64	11.5	1.0	12.5	84.1	5.2	46.1	23.9	11.8	90.0
12	3:1, 29, 200, 1, 74	10.0	1.0	11.0	72.4	7.4	43.4	19.8	15.8	72.9
13	3:1, 31, 200, 0.5, 78	7.1	1.3	8.4	70.2	11.4	46.5	16.1	26.2	75.8
14	3:1, 55, 225, 0.5, 139	7.8	1.0	8.8	74.6	7.0	40.8	14.9	14.0	70.7
15	1:1, 10, 175, 2, 24	8.4	0.5	8.9	79.5	4.0	42.9	15.8	8.5	79.2
16	3:1, 10, 175, 2, 25	13.4	0.7	14.1	73.6	4.2	45.6	26.5	9.5	78.0

^a^Experimental conditions are presented rounded for brevity in the following order: acetone/water (wt/wt) ratio, partial O_2_ pressure at reaction *T* (atm), reaction *T* (°C), reaction time (h), pressure at reaction *T* (atm)

^b^Beechwood also had extractives measured according to NREL of 11.23 wt%

Finally, for runs 3–5, the biomass was treated with 100% acetone employing N_2_, 20 vol% O_2_ and 40 vol% O_2_ (partial O_2_ pressures are shown in Table 2). Apart from a slight reduction in the lignin content, there was no significant change in the biomass content. The lack of water and the hydrolysis effect that it induces was apparent. It is, therefore, clear that water is needed, even at a small amount to initiate the hydrolysis of hemicellulose, and the cleavage of lignin-hemicellulose linkages that can lead to pronounced removal of both lignin and hemicellulose.

### Effect of O_2_ rich atmosphere

To test the effect of an O_2_ rich atmosphere, runs 6 and 7 employed a 1:1 ratio of acetone/water at 175 °C, treatment time of 2 h under an inert (run no. 6), and a 40 vol% O_2_ rich atmosphere (run no. 7). Using a mixture of acetone/water rather than the pure solvents had a significant effect, which can be clearly seen by the analysis of the pretreated biomasses (Table 2). In both cases, a synergistic effect was observed, since lignin and hemicellulose contents decreased with a consequent increase in cellulose in the resulting pulp. On one hand, the water was responsible for hydrolyzing hemicellulose, possibly disrupting its linkages with lignin achieving its partial depolymerization [6]. This allowed the acetone to solubilize the released partly depolymerized lignin, removing it from the solid biomass that, in turn, facilitated the further disruption of lignin-hemicellulose bonds. In the case of run no. 7 where the O_2_ partial pressure was higher, a further decrease in the lignin content was noted.

Lignin is a complex three-dimensional polymer with phenolic derivatives building units such as *p*-coumaryl, coniferyl, and sinapyl alcohol linked to each other by different carbon–carbon and ether linkages [21]. These have been found to be very reactive under wet oxidation conditions, making lignin a reactive molecule [10]. Ether linkages are broken more easily under oxidative conditions, depolymerizing lignin to lower molecular weight (MW) oligomers that may be dissolved much easier by solvents like acetone. Changing the acetone/water ratio to 3:1 (run no. 8) had similar effects. Again, both lignin and hemicellulose decreased; however, the lower water concentration resulted in decreased hydrolysis of hemicellulose that, in turn, affected lignin solubilization. It should be mentioned that O_2_ partial pressure was much lower (40 vol%,) under low final pressure of around 20 atm; therefore, the oxidation conditions were not very severe. Typically, 100% O_2_ gas is used to enhance delignification and maintain a low overall pressure, similar to acetone/water oxidation for the delignification of *Paulownia* spp. [22]. To enhance the oxidative effect of O_2_ atmosphere, it was decided to raise the pressure at the reaction temperature at 58 atm, which corresponded to around 24 atm of O_2_ (40 vol% O_2_) partial pressure. Using a mixture of N_2_/O_2_ rather than pure O_2_ has the added benefit of employing a lower cost gas but may result in a need for increased pressure. In future work, a techno-economic analysis will reveal the best case scenario, still it is very promising that delignification is so effective even with a N_2_/O_2_ mixture.

### Effect of acetone on water ratio

In addition to the above, the effect of acetone-to-water ratio on hemicellulose hydrolysis and removal of lignin was investigated (runs 9–11). The pressure under the reaction conditions increased to 58–64 atm (corresponding O_2_ partial pressure was 23–25 atm) to enhance the oxidative effect as explained above. Run no. 10, which employed the 1:1 acetone/water ratio, had a significant decrease in both lignin and hemicellulose with 2.2 and 10.8 wt%, respectively, in the resulting pulp. This corresponded to more than 90% of lignin removal. The resulting pulp had a cellulose content of 85.9 wt%, making it a very good feedstock for downstream enzymatic processes. Compared to run no. 7 where the low pressure of ~25 atm was used, the difference in the delignification efficiency was significant and is attributed to the increased partial pressure of the O_2_ that enhanced the depolymerization of lignin. This is in accordance to what has been reported in literature for wet oxidation process, where pure water is used as solvent. Martín and Thomsen [23] treated sugarcane, rice, cassava, and peanuts residues and concluded that an increase in O_2_ pressure resulted in higher delignification. Arvaniti et al. pretreated rape straw by wet oxidation and also found that increasing the O_2_ pressure removed more lignin overall from the solid pulp and also had a positive effect in the downstream enzymatic process [24].

**Fig. 1:**
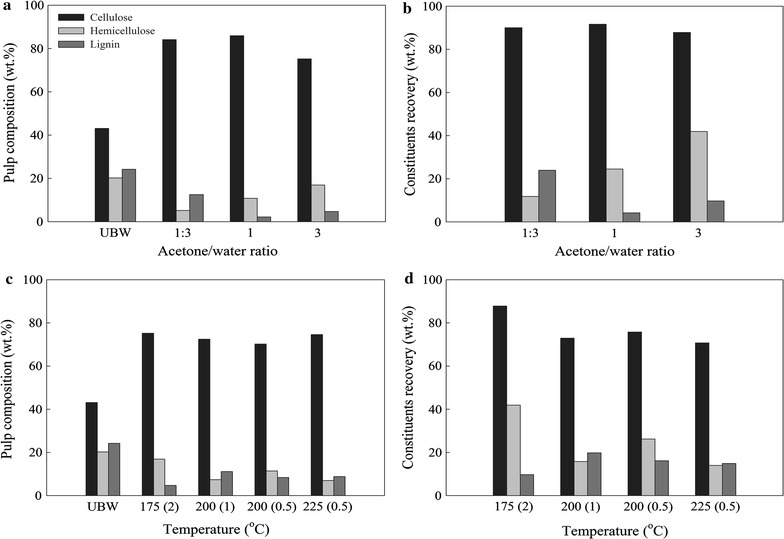
Effect of acetone/water ratio and temperature on pulp composition and constituents cellulose (*black bar*), hemicellulose (*light grey bar*), and lignin (*dark grey bar*) recoveries. **a** Pulp composition vs acetone/water ratio; untreated BW (UBW) included for comparison, **b** cellulose, hemicellulose, and lignin recoveries in solid pulp vs acetone/water ratio, **c** pulp composition vs temperature; *numbers in parentheses* are reaction time in h, **d** cellulose, hemicellulose, and lignin recoveries in solid pulp vs temperature

Acetone/water ratio of 3:1 was also used (run no. 9). A slight increase in the overall lignin content and an even bigger increase in the hemicellulose content were noted. The decrease in water content in the solvent mixture resulted in a decreased hemicellulose hydrolysis, which, in turn, also affected the lignin solubilization, even though the acetone content in the solvent mixture increased. On the other hand, in run no. 11, the acetone/water ratio of 1:3 had the opposite effects. Specifically, the high water content in the solvent mixture resulted in a more efficient hydrolysis of the hemicellulose, while the decrease in the acetone content of the mixture reduced the delignification efficiency. Decreasing the acetone content in the solvent mixture resulted in a system behaving similarly to the case of wet air oxidation where only water is used as solvent. In this case, it is the hemicellulose that is most readily hydrolyzed, while the lignin is solubilized but not as effectively as in the AWO where acetone/water ratios are 1:1 or 3:1. Typical lignin removal for wet oxidation has been found to be up to 50 wt% with the mechanism being that of an attack on the double bonds of the phenolics present in lignin and the ether bonds [25, 26]. The addition of acetone in run no. 11 led to an increase in DD to 76 wt%, compared to wet air oxidation. Therefore, the acetone played a key role in increasing the delignification efficiency even more and it appeared that an optimum was reached at the 1:1 ratio (Fig. 1).

### Effect of temperature and pretreatment time

To investigate the effect of temperature, runs 12, 13, and 14 investigated higher temperatures of 200 and 225 °C. The ratio of acetone/water was 3:1 for all runs. Due to the increase in temperature and consequently in pressure (Table 1), the reaction time decreased to 1 and 0.5 h asserting that cellulose would not be degraded. The pulp resulting from run no. 12 at 200 °C and 1 h reaction time had a lower hemicellulose content compared to run no. 9 at 175 °C and 2 h. Hence, the hemicellulose was more efficiently hydrolyzed. Still, the lignin content increased significantly from 4.7 wt% for run no. 9–11.1 wt% for run no. 12. Decreasing the reaction time to 0.5 h, the hemicellulose content increased as expected, since less time was given for the system to hydrolyze it. However, the lignin content decreased, indicating a shift in the delignification mechanism. Hayn et al. and Saddler et al. have found that treating biomass with wet oxidation at 200 °C or more resulted in the decrease of the enzymatic hydrolysis of the resulting pulp. This was attributed to a partial melting of the lignin and coating of the cellulose [27, 28]. The reduced time in run no. 13 resulted in better DD, possibly because the lignin was not allowed to repolymerise on the pulp. Finally, run no. 14 employed the short reaction time of 0.5 h at 225 °C. The DD was not altered significantly; however, the elevated temperature resulted in a decrease in the hemicellulose and hence an overall increase in the cellulose content of the pulp.

### Two-stage treatment

In an effort to maximize the DD while maintaining a high cellulose recovery in the produced pulp, a two-stage pretreatment was also tested. Essentially, the biomass was first hydrolyzed to achieve hemicellulose hydrolysis under the conditions of run no. 1. This substrate was then treated at two different AWO conditions corresponding to runs 7 and 8 (Table 1). The combination of the aforementioned conditions resulted in runs 15 and 16. The pulps produced had low hemicellulose content, while lignin was 9 and 14 wt%, respectively. Runs 7 and 8 did not remove lignin and hemicellulose efficiently, mainly due to the low O_2_ partial pressure used. In the case of the two-stage process, the removal of both lignin and hemicellulose improved significantly. Cellulose recovery is deemed to be satisfactory for a two-stage process at 80 wt% on initially available cellulose. Still, comparing the two-stage process runs, the single-stage pretreatment can remove both lignin and hemicellulose more efficiently, while maintaining high cellulose recovery (run no. 10, 91.6 wt%) under optimal conditions. Gong et al. found that hot water extraction (HWE) carried out prior to AWO treatment was very favorable for *Paulownia tomentosa* and *Paulownia elongata* biomass, which is in accordance with our results with respect to DD and hemicellulose removal [22]. Gong et al. attributed this beneficial effect to changes in the physicochemical structure of wood, such as increase in porosity, lower MW of residual lignin, and a weaker association between lignin and carbohydrates in the extracted wood. On the other hand, it has been reported that increasing the pretreatment severity of HWE may lead to lignin reacting with other degradation products [29, 30]. In addition, Ko et al. found that by increasing the pretreatment time of HWE, the acid insoluble lignin (AIL)/acid soluble lignin (ASL) ratio increased indicating changes in the lignin’s chemical structure [31]. The main drawback in the case of the two-stage pretreatment is a decrease in the cellulose recovery.

### Pulp and lignin quality

Apart from cellulose, hemicellulose, and lignin contents measured in the resulting pulps, the crystallinity index (CI) of select pulps was also measured. Specifically, pulps received from runs nos. 9, 10, and 11, were found to have CIs of 74.2, 78.7, and 74.7, respectively. Obviously, the high cellulose content found in all pulps resulted in a very crystalline material. The pulp from run no. 10, which had the highest cellulose content of 85.9 wt%, which also had the highest CI. The pulp resulting from run no. 11, which also had a high cellulose content of 84.1 wt%, had similar CI with the pulp resulting from run no. 9, which had cellulose content of 75.2 wt%. This was attributed to the higher lignin content of pulp no. 11 that is amorphous.

In addition, lignin was recovered from the acetone/water solvent mixture of several different runs. This was done via vacuum distillation. By evaporating and removing acetone, the dissolved lignin precipitated within the water. It was then filtered, washed with distilled water, and air dried for 24 h at 80 °C.

The lignin was then analyzed by the NREL methods to assess its purity. Table 3 presents the analysis of three different lignins from runs 7, 9, and 10. It was found in all cases that the recovered solids were essentially pure lignin (>90 wt%) with minimum amounts of glucan and xylan, although it should be pointed out that the NREL method cannot distinguish between lignin and pseudo-lignin. For this reason, the received lignin from run no. 10 was analyzed by FTIR and compared to a lignin received from the same biomass but through the Milox process [32]. The FTIR graphs are presented in Additional file 1.

**Table 3:** NREL analysis on acetone/water oxidation recovered lignins of beechwood

Run no.	AIL (wt%)	ASL (wt%)	Glucan (wt%)	Xylan (wt%)	Total (wt%)
7	83.3	2.5	0	2.5	88.3
9	84.6	6.7	0.1	0.8	92.2
10	88.3	3.8	0.1	0.5	92.7

Treatment with the Milox process resulted in significant degrading of the recovered lignin, indicated by the lack of peaks at characteristic wavelengths below 1500/cm corresponding to guaiacyl, syringyl, and some methyl- and methylene-side chains typically found at 1385, 1420, and 1463/cm. In contrast, the AWO gave a lignin that appeared to be much less degraded. This is in accordance with the work of Gong et al. [22] in which they analyzed the recovered AWO lignin with 2D HSQC NMR and concluded that the AWO lignin was a high purity and quality lignin. Future work should focus on fully characterizing the AWO lignin, since it can be easily separated from the solvent mixture and could potentially be upgraded towards added-value chemicals as part of a holistic biorefinery approach.

### Enzymatic hydrolysis and fermentation of AWOBW

Lignin removal is considered to be crucial for enhancing ethanol concentrations, not only by providing a material with high glucan content but also by rendering it more vulnerable to cellulolytic enzymes. In addition, non-productive binding of cellulase and β-glucosidase to lignin could be evaded at a great extent. Cellulolytic enzymes adsorption onto lignin is reported to have a significant effect on the enzymatic hydrolysis of lignocellulosic biomass resulting in reduced efficiency [33–36].

It was decided to test the suitability of different acetone/water oxidized biomasses for enzymatic hydrolysis and SSF. Overall, six different substrates were chosen, corresponding to runs 8, 9, 10, 11, 12, and 13. These substrates were produced over a different range of pressure, acetone/water ratio, temperature, and reaction time, and were found to have a range of cellulose, hemicellulose, and lignin contents. Studying them in comparison to the untreated material in downstream enzymatic processes will allow the evaluation of the pretreatment process from the total reducing sugars (TRS), glucose and ethanol production point of view.

### Effect of enzyme loading on the saccharification of AWOBW

Enzyme cost contribution in bioethanol production is not negligible; thus, changes should be primarily focused in decreasing enzyme loading at the process [37]. Therefore, enzyme loading effect investigation is crucial to achieve high saccharification yields without using an excess of enzyme dosage. To examine the effect of enzyme loading on glucose release (g/L), enzyme loads from 6 to 12 mg/g DM were used to hydrolyze AWOBW at 13 wt% solids content.

**Fig. 2:**
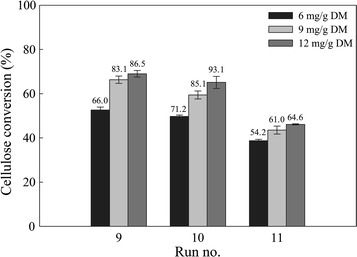
Effect of enzyme loading (Cellic® CTec2) on cellulose conversion (%) of AWOBW. Saccharification performed for 48 h at 50 °C, 100-mM citrate–phosphate buffer pH 5.0, and enzyme loadings of 6 (*black bar*), 9 (*light grey bar*), and 12 (*dark grey bar*) mg/g DM. Final glucose concentrations (g/L) are presented on *top of the bars*

Figure 2 presents the effect of enzyme loading on cellulose conversion (%) of AWOBW runs 9, 10, and 11. These samples exhibit cellulose content among the highest of all AWOBW runs; samples 9 and 10 mainly differ in respect to hemicellulose content, while sample 11 has 3–5 times higher lignin content compared to the others.

A decrease in glucose concentration of 4, 9, and 6% was noted for runs 9, 10, and 11, respectively, at 9 mg/g DM enzyme loading comparing to that of 12 mg/g DM. The use of 6 mg/g DM of enzyme loading led to a further decrease in glucose release of 27 (run 9), 21 (run 10), and 13% (run 11). Therefore, the glucose concentration difference was much lower between enzyme loadings of 9 and 12 mg/g DM than that between 6 and 9 mg/g DM. Hence, even though the enzyme loading of 12 mg/g DM resulted in the highest glucose releases after 48 h (86.5, 93.1, and 64.6 g/L from runs 9, 10, and 11, respectively) and cellulose conversions (69.0, 65.1, and 46.1%), the enzyme loading of 9 mg/g DM was selected for the experiments of enzymatic saccharification and SSF of AWOBW samples.

Comparing the release of glucose after 48 h between runs 9, 10, and 11, regardless of the enzyme loading, it is noted that run 11 had the lowest, while run 10 presented the highest glucose release in all cases. The AWOBW pulp used in run 11 had the same cellulose content as run 10, about half the content in hemicellulose and almost five times higher lignin content (Table 2). It would seem, therefore, that the critical factor in glucose release is the lignin content rather than the hemicellulose content. This is also confirmed by the release of glucose in the case of run 9, which actually has a lower cellulose content, a higher hemicellulose content but also about three times less lignin content compared to run 11. Therefore, to achieve high glucose release and cellulose conversion, the lignin content should be minimized.

**Fig. 3:**
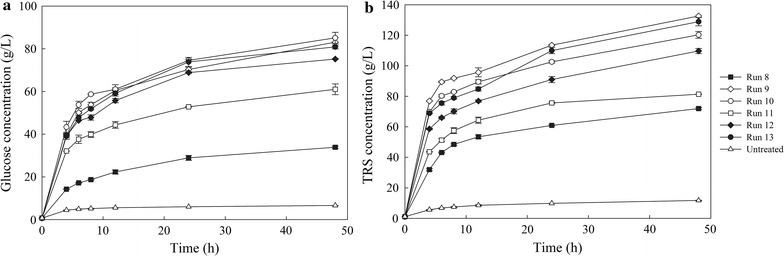
Comparison of **a** glucose and **b** TRS concentrations (g/L) during enzymatic hydrolysis of AWOBW runs 8 (*black square*), 9 (*white diamond*), 10 (*white circle*), 11 (*white square*), 12 (*black diamond*), 13 (*black circle*), and untreated BW (*white triangle*) at 13 wt% solids content, 50 °C, 100-mM citrate–phosphate buffer pH 5.0 and an enzyme loading of 9 mg/g DM

Furthermore, enzymatic hydrolysis of AWOBW samples, corresponding to runs 8, 12, and 13, was performed to screen substrates pretreated over a range of different conditions, with respect to TRS and glucose concentrations (g/L). Enzymatic hydrolysis of all six different substrates (runs 8–13) was conducted using the selected enzyme loading of 9 mg/g DM at 13 wt% solids content. The data in Fig. 3 show that the highest glucose concentration was still obtained by AWOBW run 10 (85.10 g/L) and the lowest by run 8 (33.88 g/L) after 48 h of enzymatic hydrolysis. A maximum of a 13-fold increase in glucose concentration was noted when it was compared to that achieved by untreated BW, highlighting the effectiveness of AWO.

### Evaluation of AWOBW for the production of bioethanol

Screening of AWOBW samples, corresponding to runs 8–13, with maximum ethanol concentration (g/L) as a response, was conducted to determine the AWO conditions that lead to the highest ethanol concentration in the fermentation broth. The screening experiments were carried out on small scale in 100-mL Erlenmeyer flasks at selected enzyme loading (9-mg/g DM) employing SSF process with a 12-h prehydrolysis step at 14.5 wt% solids content.

**Fig. 4:**
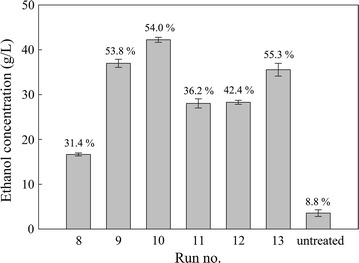
Screening of AWOBW runs 8–13 for maximum ethanol concentration (g/L) as a response factor obtained by SSF with a 12-h prehydrolysis step at 14.5 wt% solids content using an enzyme loading of 9 mg/g DM. Ethanol yields (%) are presented on *top of the bars*

As shown in Fig. 4, the AWOBW run 10 resulted in a maximum ethanol concentration of 42.2 g/L (54% ethanol yield), which is also the highest achieved among the tested samples. In addition to that, run 10 exhibited high productivity of 1.21 g/L/h after the first 24 h. The ethanol concentration for runs 8–13 followed the same trend noted for glucose release, indicating once more that lignin content was the crucial parameter in achieving high ethanol concentration and yield. Combining the high ethanol concentration and productivity exhibited by AWOBW run 10 SSF, these conditions were selected for the pretreatment of beech wood employing large-scale liquefaction/saccharification and fermentation experiments at high-solids content. Moreover, it is noteworthy that the high ethanol concentration of 42.2 g/L was achieved employing shaking flasks with known issues emerging from operating at high-solids content. This fact corroborates the enzymatic hydrolysis results where AWO rendered BW a highly digestible material for utilization as a feedstock in ethanol production processes.

### Effect of solids content on cellulose conversion

**Fig. 5:**
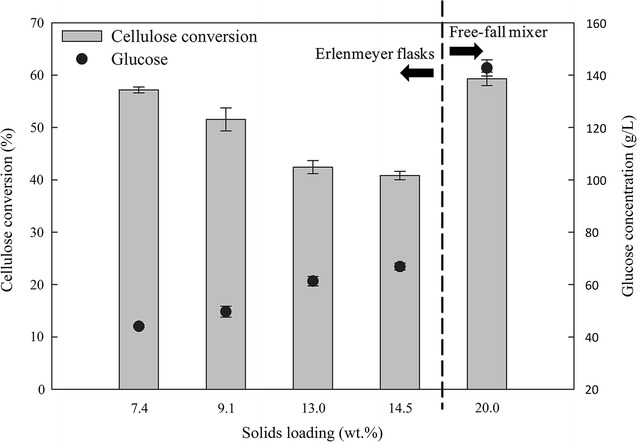
Effect of solids content on cellulose conversion (%) and glucose concentration (g/L) of AWOBW run 10 after 12 h of enzymatic hydrolysis at small scale (Erlenmeyer flasks; solids content 7.4–14.5 wt%) and large scale (free-fall mixer; solids content 20 wt%) using an enzyme loading of 9 mg/g DM

Bioconversion of AWOBW from run no. 10 at high-solids content was employed to achieve high ethanol concentrations. However, enzymatic hydrolysis at such conditions is not trivial as with increasing solids content, cellulose conversion decreases, mostly due to inadequate mixing. The effect of the initial solids concentration on glucose release and cellulose conversion using shaking flasks (7.4, 9.1, 13 and 14.5 wt%) and the free fall mixer (20 wt%) is presented in Fig. 5. Cellulose conversion and initial solids content seem to follow a linear correlation with negative slope when enzymatic hydrolysis took place in shaking flasks, exhibiting from 57.2% conversion at 7.4 wt% to 40.8% conversion at 14.5 wt% after 12 h of enzymatic hydrolysis. This underlines the negative effect of increasing solids content to cellulose conversion (calculated on a glucose release basis) when enzymatic saccharification takes place using the same stirring technique. Similar results were obtained by the previous researchers, where also a linear correlation between conversion and solids content was exhibited [38–40]. In this study, the cellulose conversion increased to 59.3% even at substantially higher initial solids content (20 wt%) when the liquefaction/saccharification step was performed in the custom free-fall mixer, rendering it an important tool for handling slurries with high-solids content. These findings are in accordance with those previously reported in literature where the use of novel stirring systems resulted in enhanced sugar yields [41].

### Fermentation of liquefacted AWOBW at high-solids content

**Fig. 6:**
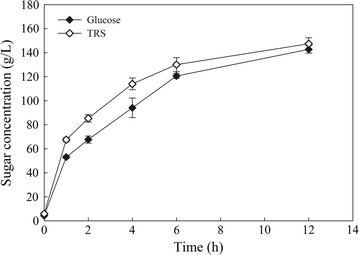
Time course of TRS (*white diamonds*) and glucose (*black diamonds*) concentration (g/L) during the liquefaction/saccharification step of AWOBW run 10 at 20 wt% solids content employing the free-fall mixer

AWOBW acquired from run no. 10 underwent a liquefaction/saccharification step at high-solids content (20 wt%) employing the free-fall mixer that was described in the “Methods” section (Additional file 2). TRS and glucose concentration (g/L) were determined during the liquefaction/saccharification step (duration of either 6 or 12 h) and the results are shown in Fig. 6. After 6 h of liquefaction/saccharification, the concentration of glucose and TRS was 120.56 ± 1.90 and 130.05 ± 5.86 g/L, respectively, while higher enzymatic hydrolysis was achieved after 12 h resulting in 142.75 ± 3.10 and 147.41 ± 4.99 g/L of glucose and TRS, respectively. Conversion of cellulose (%) based on glucose release was 55.1% after 6 h and 65.3% after 12 h of enzymatic treatment. The results suggested that a 6-h liquefaction/saccharification step seems to be adequate for the subsequent fermentation process as the glucose release (g/L) exceeded 80 g/L, which theoretically is the minimum to achieve the ethanol concentration for a low-cost distillation [13].

Moreover, the decrease in slurry’s viscosity, consisting of 20 wt% AWOBW, during the liquefaction/saccharification step was measured using an oscillatory viscometric technique with a parallel roughened plate system. The initial apparent viscosity was found to be 1.4 kPa s and rapidly decreased to 0.2 kPa s after 2 h of enzymatic hydrolysis remaining fairly stable until the end of the liquefaction/saccharification step. The decrease of 86.4% in viscosity in only 2 h shows the potential of AWOBW to be used effectively in high gravity processes.

**Fig. 7:**
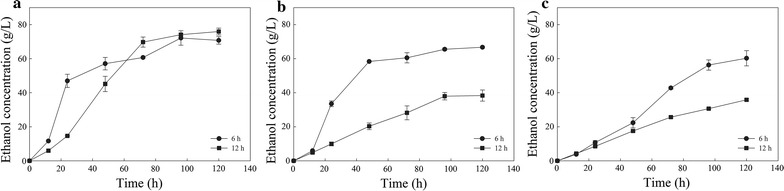
SSF performance of AWOBW run 10 **a** without the addition of extra enzyme load, **b** with the addition of enzyme loading of 4.5 mg/g DM, and **c** with the addition of enzyme loading of 9 mg/g DM, after 6 (*circle*) and 12 h (*square*) of liquefaction/saccharification step

Maximum ethanol concentration was found to be 72.2 ± 4.3 g/L (after 96 h of SSF) and 75.9 ± 2.0 g/L (after 120 h of SSF) in the case where the liquefaction/saccharification duration was 6 or 12 h, respectively (Fig. 7a). Ethanol concentration rapidly surpasses the threshold of 40 g/L, achieving 47.0 ± 3.9 g/L after 6 h of prehydrolysis and 24 h of SSF, exhibiting a high ethanol productivity of 1.96 g/L/h. However, after 12 h of prehydrolysis, even if glucose concentration is 13.3% higher than that after 6 h, ethanol concentration was found to be only 14.6 g/L (24 h of SSF), exhibiting ethanol productivity of 0.61 g/L/h. Ethanol fermentation inhibition due to high initial glucose concentration is considered to be among the bottlenecks of high gravity fermentations [15]. The ethanol production performance of a *Saccharomyces cerevisiae* strain under different substrate concentrations was investigated by Zhang et al. where after a critical glucose concentration of 160 g/L, the membrane fluidity decreased alongside yeast cell atrophy and organelle dehydration [42]. The 12-h liquefacted AWOBW exhibited a delay of 24 h to achieve ethanol concentration over 40 g/L (45.2 ± 4.5 g/L). Thus, according to these results, the 6-h liquefied AWOBW appears to be more appropriate for an industrial ethanol production process due to higher ethanol productivity. Furthermore, a prehydrolysis prolongation by 6 h led to a gain of final ethanol concentration by only 3.7 g/L.

The addition of extra enzyme loading (4.5 and 9 mg/g DM) prior to the SSF process was also investigated, aiming at the increase in ethanol production yield. Maximum ethanol concentration was found to be 66.7 ± 0.5 g/L after 120 h of SSF in the case of adding enzyme load of 4.5 mg/g DM for the 6-h liquefacted AWOBW, exhibiting a difference of 28.4 g/L of ethanol comparing to the 12-h liquefacted AWOBW (Fig. 7b). The addition of extra 9 mg/g DM of enzyme load resulted in a decrease in ethanol production with a final concentration of 60.3 ± 4.5 g/L in the case of the 6-h liquefacted AWOBW. When it comes to the 12-h liquefacted AWOBW, a difference of 24.4 g/L in final ethanol concentration was noted (Fig. 7c). These results indicated that enzyme addition probably led to an increase in glucose levels beyond a threshold where yeast cells exhibited low viability. Besides that, high enzyme loadings accumulated by adding extra enzyme prior to the SSF process could negatively affect cell viability due to additives that are present in commercial lignocellulolytic mixtures, such as sorbitol or glycerol [43]. These results are also in agreement with similar findings by Zhao et al. where an increase in cellulase loading from 10 to 20 FPU/g solid led to lower ethanol production rates for both batch and fed-batch SSF processes [44].

The ethanol production process that was employed, comprising a separate liquefaction/saccharification step at a custom made free-fall mixer and subsequent SSF of AWOBW at high DM loading, led to high ethanol concentrations. To investigate the implications of the current results in the framework of lignocellulosic ethanol production research, a comparison with various studies where high ethanol production under high-solids content is achieved is necessary. During this study, only the solid fraction that was obtained after a separation step of the pretreated slurry was used for bioethanol production. In this context, mainly works where solid fraction was used for enzymatic saccharification and ethanol fermentation are presented (Table 4) and in the majority of cases, solids content is 20 wt% or more. Nevertheless, high ethanol concentrations up to 71.4 g/L have been reported when the whole pretreated slurry was exploited for ethanol fermentation [45].

**Table 4:** Comparison of bioethanol production from various kinds of lignocellulosic biomass at high-solids content

Biomass	Solids content (wt%)	Pretreatment method	Enzyme loading (FPU/g DM)	Ethanol concentration (g/L)	Ethanol productivity (g/L·h)	Ethanol yield^f^ (%)	References
Beechwood	20.0	Acetone/water oxidation	8.4	75.9	0.63	68.1	This study
Bermudagrass	36.0	Phosphoric acid–acetone	25.0^d^	56.1	0.58	65.1	[53]
Corncob residue	20.0	Dilute acid hydrolysis-alkaline extraction	15.0	75.1	1.25	89.4	[54]
Corn stover	15.5	CELF^a^	3.1	58.8	0.49	89.2	[55]
Corn stover	20.0	Steam explosion	17.7	59.8	0.31	77.2	[40]
Corn stover	24.0	Acetic acid-catalysed hydrothermal	17.4	41.9	0.44	51.3	[16]
Eastern redcedar	20.0	Acid bisulfite	31.2	52.0	1.24	67.6	[56]
Empty palm fruit bunch	30.0	Alkali	15.0	62.5	0.66	70.6	[57]
Miscanthus	25.0	CHEMET^b^	17.0	69.2	1.24	87.2	[58]
Rapeseed straw	16.7	Dilute acid	15.0	39.9	1.67	46.7	[59]
Reed	36.0	Phosphoric acid-acetone	25.0^d^	69.3	0.72	74.7	[53]
Rice straw	13.8	Dilute acid-dilute alkali	30.0	58.7	0.49	73.4	[60]
Spruce	20.0	–^c^	7.5	40.0	0.42	53.0	[61]
Sugarcane bagasse	20.0	Formiline	10.0	80.0	0.55	82.7	[44]
Sweet sorghum bagasse	18.0	Hydrothermal	20.0	47.9	2.18	70.4	[17]
Wheat straw	25.0	Steam explosion	15.0	58.6	0.73	56.9	[46]
Wheat straw	26.0	Hydrothermal	7.0^e^	58.0	0.48	75.0	[19]

^a^Co-solvent enhanced lignocellulosic fractionation

^b^ChangHae Ethanol Multi ExTruder

^c^Provided by SECAB-E-Technology AB (Örnsköldsvik, Sweden)

^d^FPU/g cellulose

^e^Enzymes from *Fusarium oxysporum* corresponding to 1.23 FPU/g DM was also added to SSF

^f^Calculated as percentage of maximum theoretical yield

It is worth mentioning that several of the studies that are presented in Table 4 include media sterilization and/or nutrient addition, which boosts final ethanol concentrations, but on the other hand contributes to a final process cost increase. Furthermore, to enhance ethanol production, the use of enzymes such as laccases has been reported. Alvira et al. produced 58.6 g/L ethanol from steam exploded wheat straw at 25 wt% solids content when prehydrolysis step supplemented with laccase, which led to a significant final ethanol concentration increase [46].

**Fig. 8:**
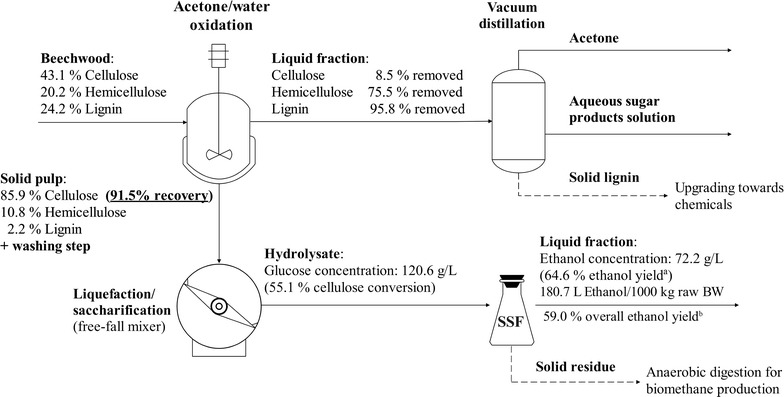
Process configuration of AWO pretreatment followed by 6-h high gravity enzymatic liquefaction/saccharification, and SSF of BW sawdust. Ethanol yields (%) were estimated for the SSF process^a^ and overall process^b^ based upon starting glucans. *Dashed lines* represent possible future stream utilization

Considering the studies that were mentioned, in most cases, enzyme loadings above 10 FPU/g DM are being used with an average of about 15 FPU/g DM. In the current study, ethanol concentrations up to 76 g/L (corresponding to ethanol yields up to 68.1%) were achieved using a relatively low enzyme loading of 8.4 FPU/g DM. In fact, as ethanol concentrations that were obtained are much higher than the threshold for economical downstream processing, even lower enzyme loadings could be employed and possibly alleviate yeast inhibition caused by glucose, resulting in improved productivity. When it comes to ethanol productivity, values ranging from 0.27 to 1.04 g/L/h were reported for most of the cases. At maximum ethanol concentrations, which were obtained from AWOBW, high productivities were determined for both the 6- and 12-h liquefacted/saccharified biomass (0.75 and 0.63 g/L/h, respectively). Furthermore, the 6-h liquefacted/saccharified AWOBW exhibited a productivity of almost 2.0 g/L/h after the first 24 h of fermentation, having already exceeded 40 g/L by that time. It can, therefore, be concluded that this study provides an effective strategy to turn BW into a highly digestible feedstock for the subsequent ethanol production process that results in high final concentrations of bioethanol. Figure 8 summarizes the entire process along with individual process step yields and products, as it was presented in this work.

## Conclusions

In the present work, the potential of a woody biomass, specifically beechwood, for the production of cellulosic ethanol was investigated, through the optimization of the acetone/water oxidized pretreatment and SSF process. The optimal pretreatment conditions were acetone/water ratio 1:1, 40 atm initial pressure of 40 vol% O_2_ gas (20 °C) and 64 atm at reaction temperature of 175 °C for 2 h incubation. These pretreatment conditions allowed the isolation of lignin, which was found to be intact and could, therefore, potentially lead to high-added-value products, such as phenols and aromatics in a holistic biorefinery approach. The subsequent liquefaction and saccharification process of the pretreated BW feedstock at high-solids content allowed the production of high ethanol concentration (75.9 ± 2.0 g/L). To the authors’ knowledge, the obtained ethanol concentration is the highest reported in literature utilizing BW residual biomass, underpinning the potential of the pretreatment and fermentation process followed for the efficient conversion of biomass to ethanol and bio-based chemicals.

## Methods

### Raw materials

Lignocellulosic biomass used as a feedstock in the experiments of the current study was a commercially available beech wood (BW) with particle size 150–500 μm (Lignocel® HBS 150-500) and was handled, as described by Kalogiannis et al. [32].

### Strains and enzymes

*Saccharomyces cerevisiae* strain Ethanol Red®, developed for the industrial ethanol industry by Fermentis (Marcq-en-Barœl, France) exhibiting high ethanol tolerance and cell viability during HG fermentation, was employed in SSF experiments. Commercial enzyme solution Cellic® CTec2 was obtained from Novozymes A/S (Bagsværd, Denmark) and used for the liquefaction and saccharification of acetone/water oxidation pretreated beech wood (AWOBW). Filter paper activity was determined according to Ghose [47] and found to be 84 FPU/mL. Protein content was measured using the Bradford assay [48] and was 90 mg/mL. All other chemicals and reagents were of analytical grade.

### Acetone/water oxidation pretreatment

AWO of biomass was carried out in a Hastelloy C-276 Parr autoclave with a volume of 975 mL. 50 g of solid feedstock were fed into the reactor and 500 g of an acetone/distilled water mixture were then poured at a ratio of liquid to solid 10:1. The reactor was tightly sealed and pressurized up to 40 atm with a N_2_/O_2_ mixture. A Parr Model 4848 reactor controller was used to control the temperature inside the reactor. Uniform heating and temperature was ensured by mixing of the suspension with a propeller type agitator rotating at 150 rpm. The temperature was set at 175 °C for a reaction time of 2 h in all cases. Reaching the desired temperature took typically 15 min; this was defined as time zero. After the prescribed reaction time, the cool down time was minimized to around 15 min by cooling the reactor with air externally and internally with water that was circulated through a cooling coil. The solid residue was filtered from the liquid phase, washed with 250 g of acetone, and dried overnight in an oven at 80 °C. A round of wash with distilled water and dry overnight was followed.

Among the parameters studied were the pressure, reaction time, temperature, and acetone-to-water ratio. Specifically, two different pressures were employed. The autoclave was pressurized at low pressure (LP) of 8.5 atm and at high pressure (HP) of 40 atm at 20 °C. Final pressure depended on the reaction temperature. The temperatures studied were 175, 200, and 225 °C for reaction times of 2, 1, and 0.5 h, respectively. In addition, the biomass was treated hydrothermally with 100% water and with 100% acetone under either an inert atmosphere (N_2_) or pressurized with 40 vol% O_2_. The acetone-to-water ratio was also investigated. Apart from the runs that employed 100% water or acetone the 3:1, 1:1, and 1:3 acetone:water ratios were tested as well. All experimental conditions are presented in Table 1. All runs were repeated twice and the mean values are reported. The resulting pulps were dried and weighed, while the original biomass and the resulting pulps were analyzed by the NREL method to determine (see “Analytical methods” section) cellulose, hemicellulose, and lignin contents. Standard deviation for the recovered pulps was below ±1.5%. This allowed for the determination of the recoveries of each biomass constituent in the solid pulp. The delignification degree (DD) can be calculated as 100% lignin recovery (%).

### Analytical methods

TRS concentration was determined according to dinitro-3,5-salicylic acid (DNS) method [49] and glucose was measured according to commercial enzyme preparation of glucose oxidase/peroxidase (GOD/PAP) assay. The cellulose, hemicellulose, lignin, and ash contents of lignocellulosic biomass were determined according to the procedures provided by National Renewable Energy Laboratory (NREL; Golden, CO, USA) [50]. Ethanol produced during the SSF was analyzed by a high-pressure liquid chromatography (HPLC) apparatus consisting of a fully integrated solvent delivery system (LC-20AD; Shimadzu, Kyoto, Japan) coupled with a refractive index detector (RID 10A; Shimadzu), an auto sampler (SIL-20A; Shimadzu), and a computer-based integration system (LCsolution Version 1.24 SP1; Shimadzu). An Aminex HPX-87H (300 × 7.8 mm, particle size 9 μm; Bio-Rad, Hercules, CA, USA) chromatography column was used. Mobile phase was 5 mM sulphuric acid in degassed HPLC grade water at a constant flow rate of 0.6 mL/min and the column temperature was maintained at 40 °C using a column heater (Merck Millipore, Darmstadt, Germany).

Fourier transform infrared spectroscopy (FTIR) analysis was employed for further characterization of the lignin samples’ structure. Details may be found elsewhere [51]. X-ray diffraction analysis was done on a Siemens D500, copper ray with a Nickel filter (*λ* = 1.5406 Å, voltage 40 kV, intensity 30 mA). The angle 2*θ* was between 5° and 50° with a step 0.04 step time 2 s.

### Enzymatic liquefaction and saccharification of AWOBW

Enzymatic saccharification of AWOBW samples was carried out in 100-mL Erlenmeyer flasks (small scale) in an orbital shaker (Zhicheng, Shanghai, China). BW loadings of 13 wt% (6–12 mg Cellic® CTec2/g DM) and 7.4–13 wt% (9 mg Cellic® CTec2/g DM) in 100-mM citrate–phosphate buffer at pH 5.0 were employed for investigating the effect of enzyme loading and solids content on cellulose conversion, respectively. Saccharification was performed for 48 h at 50 °C and 200 rpm. Microbial contaminations were prevented by the addition of 0.02% (w/v) sodium azide. Samples were taken at different time intervals and soluble sugars were determined, to estimate cellulose and total polysaccharides hydrolysis. Each experiment was carried out in duplicates. Error bars in figures represent the standard deviation between experimental measurements.

AWO pretreated samples at 14.5 wt% loading underwent a liquefaction step in 100-mL Erlenmeyer flasks in an orbital shaker at 50 °C, in 100-mM citrate–phosphate buffer pH 5.0 for 12 h using 9-mg/g DM of Cellic® CTec2. After the liquefaction step, slurry temperature was adjusted to 35 °C for the subsequent fermentation process.

Liquefaction and saccharification of AWOBW at high initial DM content of 20 wt% to achieve high sugar concentration were enabled employing a free fall mixer (large scale), consisting of two vertically placed, cylindrical liquefaction chambers of 6 cm in width and a diameter of 25 cm with the ability to rotate for proper material mixing [19]. Rotation speed was adjusted at 7 rpm and was changing from clock to anti-clock wise every 2 min. The liquefaction chambers were maintained at 50 °C by an oil-filled heating jacket. Enzyme load was 9-mg/g DM of Cellic® CTec2 at 100-mM citrate–phosphate buffer pH 5.0. The duration of liquefaction-saccharification step was either 6 or 12 h.

### Viscosity measurements

The liquefaction step of AWOBW catalysed by Cellic® CTec2 was carried out in the free-fall mixing apparatus described previously. For the determination of the viscosity, aliquots of the liquefacted AWOBW were taken in different time intervals and apparent viscosities of slurries were measured with an Anton Paar Physica MCR rheometer (Anton Paar Gmbh, Styria, Austria), as described previously [16]. Apparent viscosities during enzymatic hydrolysis were compared at shear rate of 0.03/s (*ω* of 60 rad/s). The parallel plates’ diameter was 25 mm and the gap between them was ≈2 mm.

### Simultaneous saccharification and fermentation experiments

Fermentations of non-sterilized liquefacted AWOBW at 14.5 (small scale) and 20 wt% DM (large scale) were performed in 100-mL Erlenmeyer flasks at pH 5.0 and temperature of 35 °C in an orbital shaker (80 rpm). *S. cerevisiae* strain Ethanol Red®, corresponding to 15 mg/g DM, was used for the anaerobic fermentation without the addition of extra nutrients in the fermentation broth. Samples were taken at 0, 12, 24, 48, 72, 96, and 120 h and were analyzed for ethanol. The ethanol yield was calculated according to the method of Zhang and Bao [52] for high-solids and high ethanol concentration SSF process. Each trial was carried out in duplicates. Error bars in figures represent the standard deviation between experimental measurements.

## Additional files

**Additional file 1: Figure S1.** FTIR spectra of standard Kraft lignin, Milox [32] derived lignin and acetone/water oxidation lignin.

**Additional file 2: Figure S2.** Free-fall mixer that was employed for the liquefaction/saccharification of AWOBW run no. 10 at high-solids content (20 wt%). (a) Front view, (b) top view, (c) inside view, and AWOBW slurry (d) before and (e) after the liquefaction/saccharification step. Free-fall mixer was designed and constructed by Paschos et al. [19], and was funded by the European Community’s 7th Framework Program (Project ID 213139; the HYPE project).

## Abbreviations

AIL: acid insoluble lignin
ASL: acid soluble lignin
AWO: acetone/water oxidation
AWOBW: acetone/water oxidation pretreated beech wood
BW: beech wood
CI: crystallinity index
DD: degrees of delignification
DM: dry matter
DNS: dinitro-3,5-salicylic acid
FTIR: Fourier transform infrared spectroscopy
HCW: hot compressed water
HG: high gravity
HP: high pressure
HPLC: high-pressure liquid chromatography
HWE: hot water extraction
LP: low pressure
MW: molecular weight
NREL: national renewable energy laboratory
SSF: simultaneous saccharification and fermentation
TRS: total reducing sugars
UBW: untreated beechwood
