# Supplementary figures and images for: Production of high concentrated cellulosic ethanol by acetone/water oxidized pretreated beech wood
Source: Biotechnol Biofuels. 2017 Feb 28;10:54. doi: 10.1186/s13068-017-0737-9 (PMC5331700; doi:10.1186/s13068-017-0737-9)

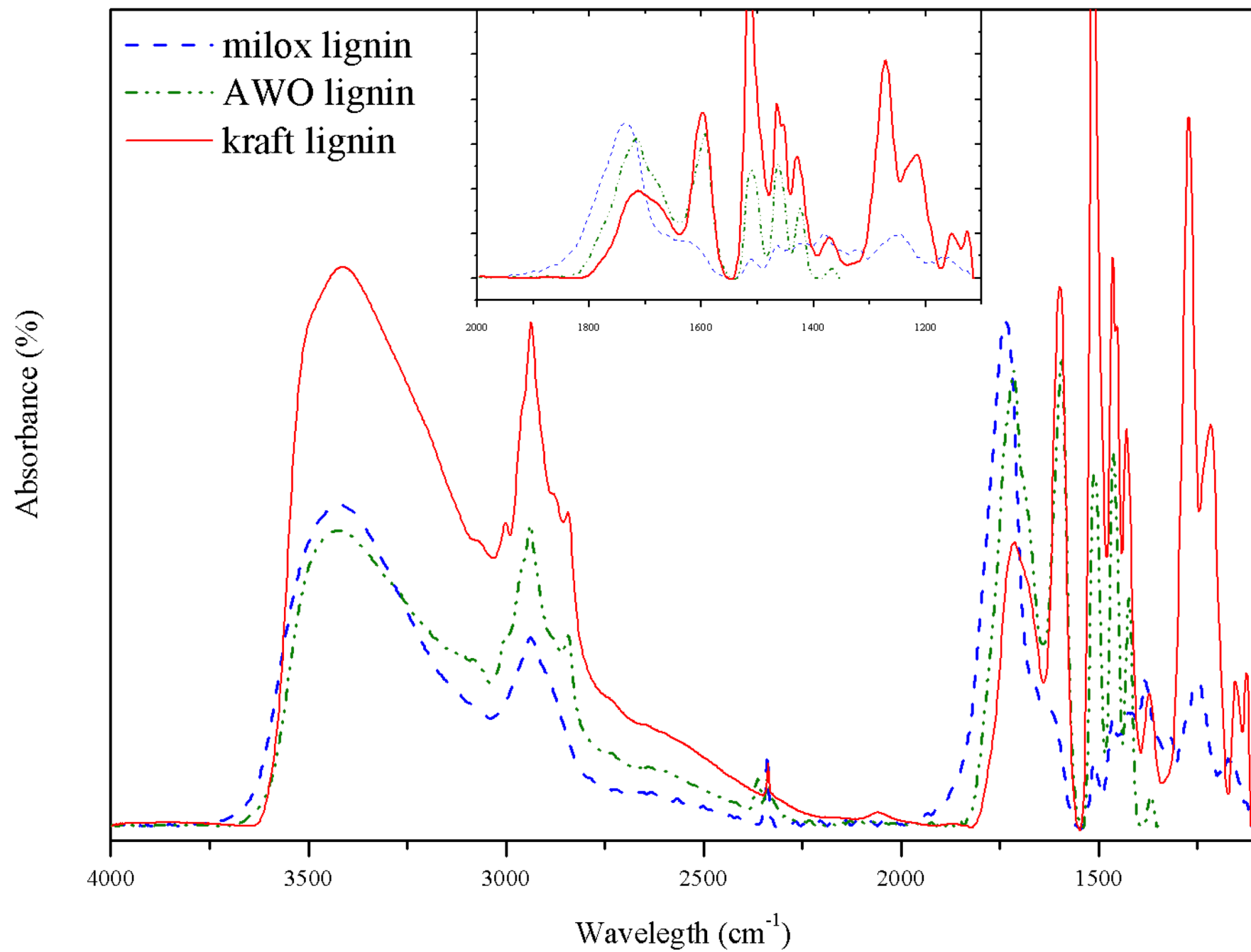

Supplement: Supplementary file 1 — Additional file 1: Figure S1. FTIR spectra of standard Kraft lignin, Milox [32] derived lignin and acetone/water oxidation lignin. [file 13068_2017_737_MOESM1_ESM.pdf]

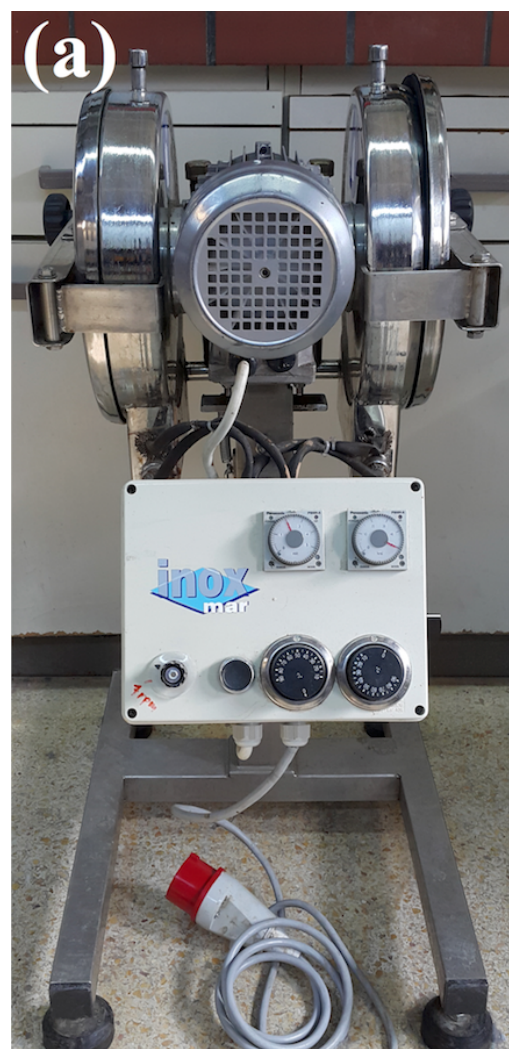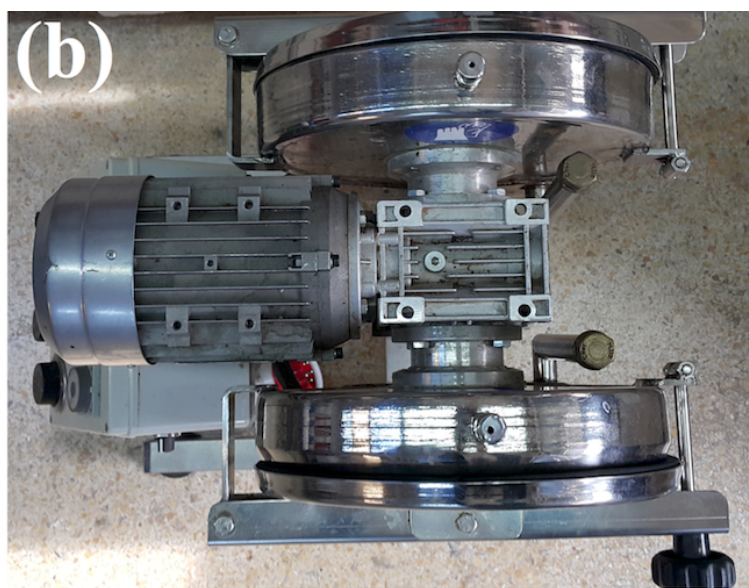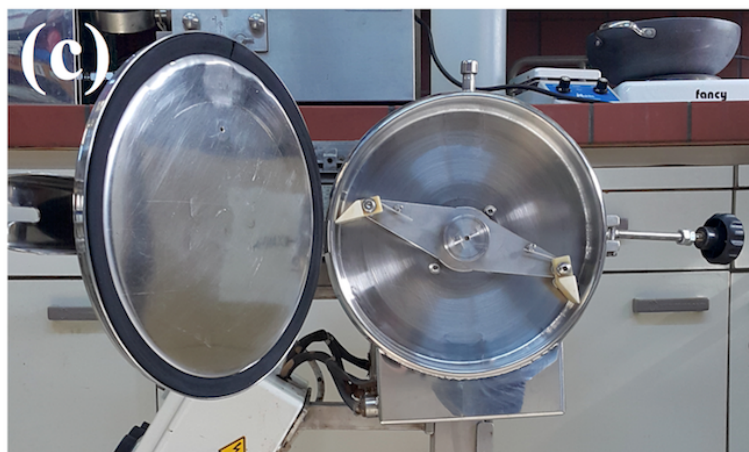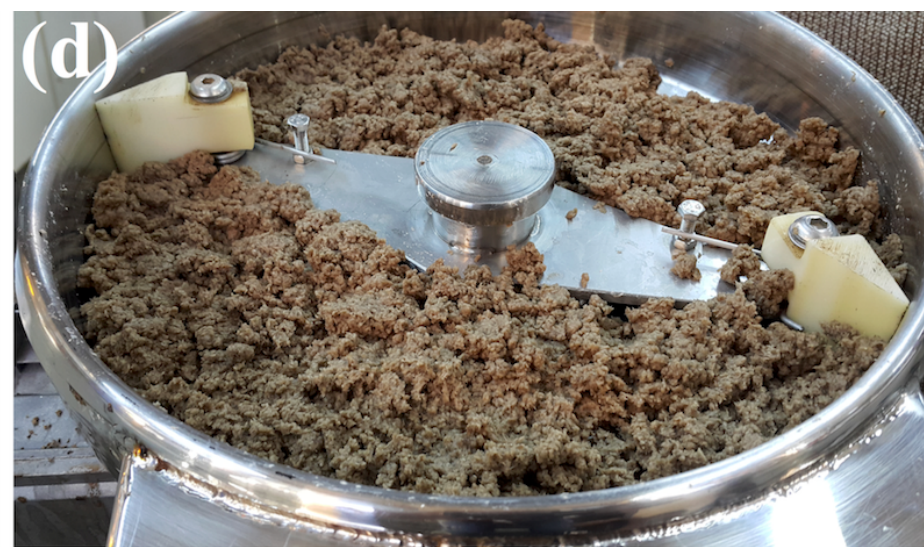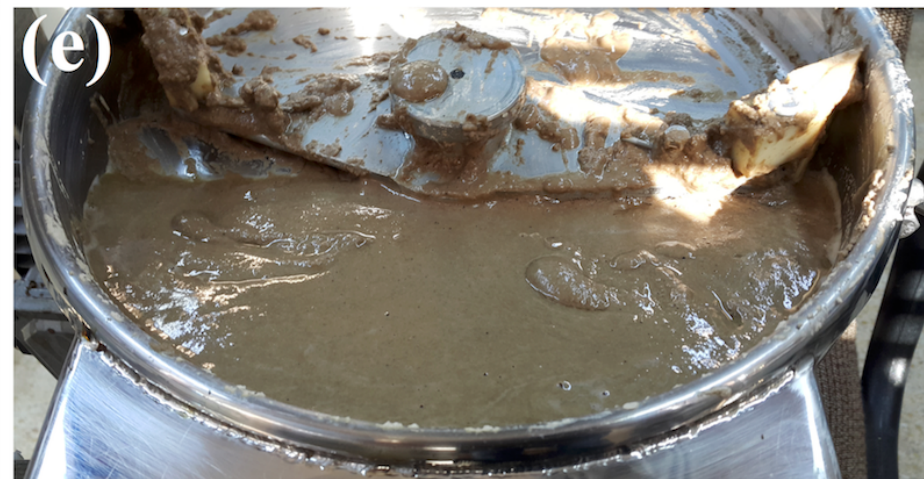

Supplement: Supplementary file 2 — Additional file 2: Figure S2. Free-fall mixer that was employed for the liquefaction/saccharification of AWOBW run no. 10 at high-solids content (20 wt%). (a) Front view, (b) top view, (c) inside view, and AWOBW slurry (d) before and (e) after the liquefaction/saccharification step. Free-fall mixer was designed and constructed by Paschos et al. [19], and was funded by the European Community’s 7th Framework Program (Project ID 213139; the HYPE project). [file 13068_2017_737_MOESM2_ESM.pdf]
